# A perfused human blood–brain barrier on-a-chip for high-throughput assessment of barrier function and antibody transport

**DOI:** 10.1186/s12987-018-0108-3

**Published:** 2018-08-31

**Authors:** Nienke R. Wevers, Dhanesh G. Kasi, Taylor Gray, Karlijn J. Wilschut, Benjamin Smith, Remko van Vught, Fumitaka Shimizu, Yasuteru Sano, Takashi Kanda, Graham Marsh, Sebastiaan J. Trietsch, Paul Vulto, Henriëtte L. Lanz, Birgit Obermeier

**Affiliations:** 1 Mimetas BV, J.H. Oortweg 19, 2333 CH Leiden, The Netherlands; 2 Department of Cell and Chemical Biology, Leiden University Medical Centre, Einthovenweg 20, 2333 ZC Leiden, The Netherlands; 3 Biogen, 225 Binney Street, Cambridge, MA 02142 USA; 4 Yamaguchi University Graduate School of Medicine, Minamikogushi, Ube, Yamaguchi 7558505 Japan

**Keywords:** Blood–brain barrier, Microfluidics, Organ-on-a-chip, BBB, Antibody transcytosis

## Abstract

**Background:**

Receptor-mediated transcytosis is one of the major routes for drug delivery of large molecules into the brain. The aim of this study was to develop a novel model of the human blood–brain barrier (BBB) in a high-throughput microfluidic device. This model can be used to assess passage of large biopharmaceuticals, such as therapeutic antibodies, across the BBB.

**Methods:**

The model comprises human cell lines of brain endothelial cells, astrocytes, and pericytes in a two-lane or three-lane microfluidic platform that harbors 96 or 40 chips, respectively, in a 384-well plate format. In each chip, a perfused vessel of brain endothelial cells was grown against an extracellular matrix gel, which was patterned by means of surface tension techniques. Astrocytes and pericytes were added on the other side of the gel to complete the BBB on-a-chip model. Barrier function of the model was studied using fluorescent barrier integrity assays. To test antibody transcytosis, the lumen of the model’s endothelial vessel was perfused with an anti-transferrin receptor antibody or with a control antibody. The levels of antibody that penetrated to the basal compartment were quantified using a mesoscale discovery assay.

**Results:**

The perfused BBB on-a-chip model shows presence of adherens and tight junctions and severely limits the passage of a 20 kDa FITC-dextran dye. Penetration of the antibody targeting the human transferrin receptor (MEM-189) was markedly higher than penetration of the control antibody (apparent permeability of 2.9 × 10^−5^ versus 1.6 × 10^−5^ cm/min, respectively).

**Conclusions:**

We demonstrate successful integration of a human BBB microfluidic model in a high-throughput plate-based format that can be used for drug screening purposes. This in vitro model shows sufficient barrier function to study the passage of large molecules and is sensitive to differences in antibody penetration, which could support discovery and engineering of BBB-shuttle technologies.

**Electronic supplementary material:**

The online version of this article (10.1186/s12987-018-0108-3) contains supplementary material, which is available to authorized users.

## Background

The blood–brain barrier (BBB) ensures a homeostatic environment for the central nervous system (CNS) and is essential for healthy brain functioning. The BBB comprises specialized endothelial cells and supporting cells, such as astrocytes and pericytes. Due to a combination of specific transport mechanisms and the presence of adherens junctions and tight junctions, the BBB controls passage of compounds into the brain [1–5]. This way, the BBB protects the brain from many harmful substances that circulate in the blood. However, the BBB’s barrier properties also complicate the treatment of CNS disorders, as many small- and large-molecule pharmaceuticals are restricted from entering the brain in quantities that are large enough to elicit a therapeutic response [6]. It is therefore necessary to develop improved drug delivery strategies that enable efficient delivery of biopharmaceuticals to the brain.

The BBB employs specialized transporter systems to allow essential nutrients to enter the brain. The transport system that is most attractive to deliver large-molecule drugs into the brain is receptor-mediated transcytosis (RMT). In RMT, a ligand (or antibody) binds a receptor on the luminal surface of a brain endothelial cell, after which it undergoes internalization via endocytosis and is trafficked to the abluminal side, where it can be released and gain access to the brain parenchyma. Harnessing this process for therapeutic drug delivery is compelling, as it could allow for selective transport into the CNS in a non-invasive manner, without disruption of the BBB [7, 8]. Several studies have demonstrated increased CNS exposure to therapeutic antibodies by combining them with RMT targeting antibodies against the transferrin receptor, insulin receptor, low-density lipoprotein receptor-related proteins 1 and 2, and the large neutral amino acid transporter 1 [9–11]. However, challenges exist in optimizing antibody properties (such as affinity, valency, bispecific format, and Fc receptor engagement) to effectively and safely traffic across the brain endothelium [12–16]. Improved in vitro models that enable further research of the cellular and molecular mechanisms underlying transcytosis at the BBB are needed to improve these CNS drug delivery technologies [14, 17, 18].

While in vivo models can be used to study an intact BBB in its physiological environment, the complexity involved in deciphering whole-organism drug distribution and the lower throughput of these studies limits their use in screening for BBB-penetrant antibodies. For this reason, in vivo research in the field is complemented by simpler and faster in vitro models, such as the Transwell method [19–22] and several on-a-chip systems [23–29]. Although the field of in vitro BBB modelling has tremendously progressed in recent years, there is a need for a model that combines fast, high-throughput readouts with physiologically relevant conditions, such as flow, co-culture, and the absence of artificial membranes.

In this manuscript, we show the development of an in vitro model of the human BBB in a high-throughput microfluidic platform. The platform allows patterning of extracellular matrix gel by means of surface tension. A blood vessel is grown adjacent to that gel and a third channel is used to insert astrocytes and pericytes. The system is free of artificial membranes, accommodates fluid flow through the blood vessels, and allows fluid-phase sampling of molecules that penetrate the endothelial and matrix layers. Using two different antibodies, we show that the model is sensitive to differences in antibody penetration of brain endothelial cells. The model may support further discovery of antibody BBB-shuttle technologies.

## Methods

### Cell culture

Cell lines of brain endothelial cells, pericytes, and astrocytes were provided by Yamaguchi University, Japan, and originate from the following human primary cell sources: human brain microvascular endothelial cells (TY10 cell line) were isolated from normal brain tissue from a patient with meningioma. Human brain pericytes (hBPCT cell line) were derived from brain tissue of a patient that died from a heart attack. Human astrocytes (hAst cell line) were generated from human primary astrocytes distributed by Lonza (Basel, Switzerland). All three cell types were immortalized with retroviral vectors harboring a SV40 large T antigen gene that is engineered to drive proliferation at 33 °C [30–34] and have been used to model the BBB in previous studies [35–38]. Cells were cultured at 33 °C, 5% CO_2_ to allow optimal cell expansion in T75 flasks (734-2705, Corning, NY, USA), which were pre-coated with 50 µg/mL collagen-I (Cultrex 3D collagen-I Rat Tail, 5 mg/mL, 3447-020-01, AMSbio, Abingdon, UK) in 1% acetic acid (A6283, Sigma, St. Louis, MO, USA) in water. TY10 cells were used between passage 17–25 and cultured in ScienCell endothelial cell medium (#1001, Sciencell, Carlsbad, CA, USA). The hAst cells were used between passage 7–12 and cultured in ScienCell astrocyte medium (#1018, Sciencell). The hBPCT cells were used between passage 14–25 and cultured in ScienCell pericyte medium (#1012, Sciencell). Cells were routinely tested for mycoplasma contamination and found negative.

### Culture of TY10 microvessels in the two-lane OrganoPlate

Two-lane OrganoPlates (Mimetas BV, the Netherlands) with 400 µm × 220 µm (w × h) channels were employed. Phaseguides had dimensions of 100 µm × 55 µm (w × h) and 2 µL of gel composed of 4 mg/mL collagen-I (Cultrex 3D collagen-I Rat Tail, 5 mg/mL, 3447-020-01, AMSbio), 100 mM HEPES (15630-122, Thermo Fisher, Waltham, MA, USA) and 3.7 mg/mL NaHCO_3_ (S5761, Sigma) was dispensed in the gel inlet and the OrganoPlate was incubated for 15 min at 33 °C. After plate incubation, 25 µL of PBS was added to the gel inlet to prevent the gel from drying out. A TY10 cell suspension of 1.5 × 10^7^ cells/mL was prepared and 2 µL was seeded in the medium inlet. 50 µL of medium was added to the medium inlet and PBS was aspirated from the gel inlet. The plate was incubated on the side for 3 h in the incubator to allow the cells to sediment against the collagen-I gel and attach. After incubation, 50 µL of medium was added to the medium outlet. The OrganoPlate was placed on an interval rocker switching between a + 7° and − 7° inclination every 8 min (Mimetas Rocker Mini, Mimetas BV), allowing bidirectional flow. Cells were cultured at 33 °C (and 5% CO_2_) to allow full cell coverage of the ECM gel. Medium was refreshed every 2–3 days. A schematic representation of all steps is shown in Additional file 1. The following media were used to assess their influence on barrier function: ScienCell endothelial cell medium (#1001, Sciencell), Cell Biologics endothelial cell medium (#H1168, Cell Biologics, Chicago, IL, USA), MV2 medium (C-22121, Bioconnect, Huissen, the Netherlands), and EBM-2 medium (cc-3156, Lonza).

### BBB co-culture in the three-lane OrganoPlate

OrganoPlate BBB co-culture was performed using three-lane OrganoPlates with 400 µm × 220 µm (w × h) channels (Mimetas BV). Phaseguides had dimensions of 100 µm × 55 µm (w × h). To establish a BBB co-culture, a collagen-I gel was dispensed in the gel inlet of the chips and filled the middle channel. TY10 cells were seeded and cultured in the top channel as described in the previous section. After 3 days, 2 µL of a 7 × 10^6^ cells/mL cell suspension of hAst and hBPCT cells (1:3 ratio) was seeded in the bottom channel. The plate was incubated on the side for 1.5 h to allow the hAst and hBPCT cells to attach to the collagen-I gel. After incubation, fresh Sciencell astrocyte medium is added to the inlet and outlets of the top channel (50 µL in each), after which perfusion is reinstated by placing the plate on the rocker platform. During the entire culture period, only the top channel, which contains the TY10 microvessel, is perfused to allow optimal endothelial barrier strength. Medium was refreshed every 2–3 days. Assays were performed at day 7. A schematic representation of all steps is shown in Additional file 2.

For the images shown in Fig. 3f, g, hBPCT cells were labeled with Calcein red™ AM (21900, AAT Bioquest, Sunnyvale, CA, USA) and hAst cells were labeled with green-fluorescent calcein-AM (C3099, Thermo Fisher) before seeding in the OrganoPlate.

### Immunocytochemistry

Cultures in the OrganoPlate were fixed with 100% methanol (− 20 °C, 494437, Sigma) and permeabilized with 0.3% Triton X-100 (T8787, Sigma). Cells were incubated with blocking solution (2% FCS, 2% bovine serum albumin (BSA, A2153, Sigma), 0.1% Tween-20 (P9416, Sigma)) for 45 min. Primary antibody was incubated for 1–2 h, after which secondary antibody was incubated for 30 min. The following antibodies were used: anti-claudin-5 (35-2500, Thermo Fisher), anti-VE-cadherin (ab33168, Abcam), anti-PECAM-1 (M0823, Dako), goat anti-rabbit AlexaFluor 488 (A11008, Thermo Fisher), goat anti-rabbit AlexaFluor 555 (A21428, Thermo Fisher), goat anti-mouse AlexaFluor 488 (A11001, Thermo Fisher), goat anti-mouse AlexaFluor 555 (A21422, Thermo Fisher), and donkey anti-mouse AlexaFluor 647 (A31571, Thermo Fisher). Nuclei were stained using Hoechst (H3570, Thermo Fisher). All steps were performed at room temperature (RT). Cells were imaged with ImageXpress Micro XLS and Micro XLS-C HCI Systems (Molecular Devices, San Jose, CA, USA).

TY10 cells grown in a collagen-I coated 24-well glass bottom plate (P24-0-N, Cellvis, Mountain View, CA, USA) were fixed with 4% paraformaldehyde (50-980-487, Thermo Fisher) for 10 min and incubated with primary antibody against the human transferrin receptor (A11130, Thermo Fisher) for 3 h, followed by 1 h incubation with goat anti-mouse Alexa Fluor 488 (A11001, Thermo Fisher). Nuclei were stained with Hoechst and cells were imaged using a Zeiss LSM 710 Confocal Microscope (Zeiss, Oberkochen, Germany).

### Barrier integrity assay

Chips were washed with culture medium (25 µL on all inlets and outlets, 1 × 5 min) to ensure proper flow profiles during the subsequent barrier integrity assay. Next, all medium was aspirated from the chips and 20 µL of medium without fluorescent compound was added to the basal side of the chips (for the two-lane OrganoPlate this is the gel inlet, for the three-lane OrganoPlate these are the gel inlets and outlets and bottom medium inlets and outlets). Medium containing 0.1 mg/mL FITC-dextran (20 kDa, FD20S, Sigma) was added to the top channel, which contained the TY10 microvessel (40 µL on inlet, 30 µL on outlet) and image acquisition was started. Leakage of the fluorescent molecule from the lumen of the microvessel into the adjacent gel channel was automatically imaged using an ImageXpress XLS Micro HCI system (molecular devices). The ratio between the fluorescent signal in the basal and apical region of the tube was analyzed using FiJi [39]. Graphs were plotted using GraphPad Prism 6 (GraphPad Software, San Diego, CA, USA).

### Analysis of cell surface binding of anti-hTfR MEM-189

TY10 cells were cultured to confluency and lifted with accutase for 1 h. 2.5 × 10^5^ cells/mL were mixed with antibody in PBS with 0.5% BSA and incubated for 1 h at 4 °C. Cells were washed 3× and incubated with 3 µg/mL PE-goat anti-mIgG (115-116-146, Jackson ImmunoResearch, Cambridgeshire, UK) for 1 h at 4 °C, then washed and fixed with 1% paraformaldehyde for 10 min at RT. Cell fluorescence intensity was analyzed by flow cytometry (FACSCalibur, BD Biosciences, Franklin Lakes, NJ, USA) and data was analyzed in FlowJo v10 (FlowJo LLC, Ashland, OR, USA) and GraphPad Prism software.

### Antibody transcytosis assay

Anti-human transferrin receptor mouse monoclonal antibody MEM-189 mIgG1 (MA1-21562, Thermo Fisher, 10 × 0.1 mg) was dialyzed into pyrogen free PBS to remove the azide in the supplied storage solution. For negative control, an anti-hen egg lysozyme (anti-HEL) antibody (F10.6.6, Genbank AF110316 VH and AY277254.1 VL) was expressed as a mouse IgG1 antibody in CHO cells and purified by recombinant protein A Sepharose (GE) affinity chromatography and size exclusion chromatography (superdex 200).

Chips were washed once with medium to ensure proper flow profiles (as described for the barrier integrity assay). Next, medium was aspirated from the chips, after which 20 µL of fresh medium without antibody was added to the gel inlets and outlets and the bottom medium inlets and outlets. A total of 70 µL of antibody dilution (1.25 µM in medium) was added to the top channel and the OrganoPlate was incubated on the rocker platform in the incubator for 1 h after which basal samples were taken, which consisted of the full contents of the gel inlet and outlet and bottom medium inlet and outlet.

### Analysis of antibody contents in basal samples

A Meso Scale Discovery (MSD) platform based quantitative immunoassay was used to determine the concentration of antibodies in basal samples collected from the OrganoPlate. Multi-Array 96-well MSD high-binding plates (L15XB-3/L11XB-3, Meso Scale Discovery, Rockville, MD, USA) were coated overnight at 4 °C with 5 µg/mL capturing agent AffiniPure goat anti-mouse IgG, Fcγ fragment specific (115-005-071, Jackson ImmunoResearch). Plates were blocked (1% BSA in PBS) for 2 h, after which 100 µL/well standards (generated from 1.25 µM antibody stock dilutions) or samples were added to the plate and incubated for 1.5–2 h. Plates were incubated with 100 µL/well 0.25 µg/mL primary detection agent Biotin-SP-conjugated AffiniPure goat-anti-mouse IgG (115-005-071, Jackson ImmunoResearch) for 45 min, followed by 100 µL/well 0.75 µg/mL secondary detection agent MSD Sulfo-TAG StreptAvidin (R32AD-1, Meso Scale Discovery) for 30 min. Between each step, the plates were washed 4× with wash buffer (PBST: 1× PBS with 0.05% Tween-20, 28352, Thermo Fisher). Immediately before MSD read, 2× Read Buffer (MSD Read Buffer T (4×), R92TD-2, diluted 1:2 in diH_2_O) was added to the plates. Plates were read on a MSD QuickPlex SQ 120 in automatic read mode, then a Sigmoidal, 4PL, X = log(concentration) interpolation was used to determine antibody concentration in GraphPad Prism 7.02 (GraphPad Software). The apparent permeability (P_app_) of both antibodies was determined using the following formula: P_app_ = [ΔC_receiver_/Δt] × [V_receiver_/(A_barrier_ × C_donor, initial_)], in which ΔC_receiver_/Δt is the change in antibody concentration in the receiving compartment over time, V_receiver_ is the volume of the receiving compartment, A_barrier_ is the surface area of the barrier, and C_donor, initial_ is the initial antibody concentration of the donor compartment.

## Results

### Perfused microvessels of TY10 brain endothelial cells in the two-lane OrganoPlate

**Fig. 1:**
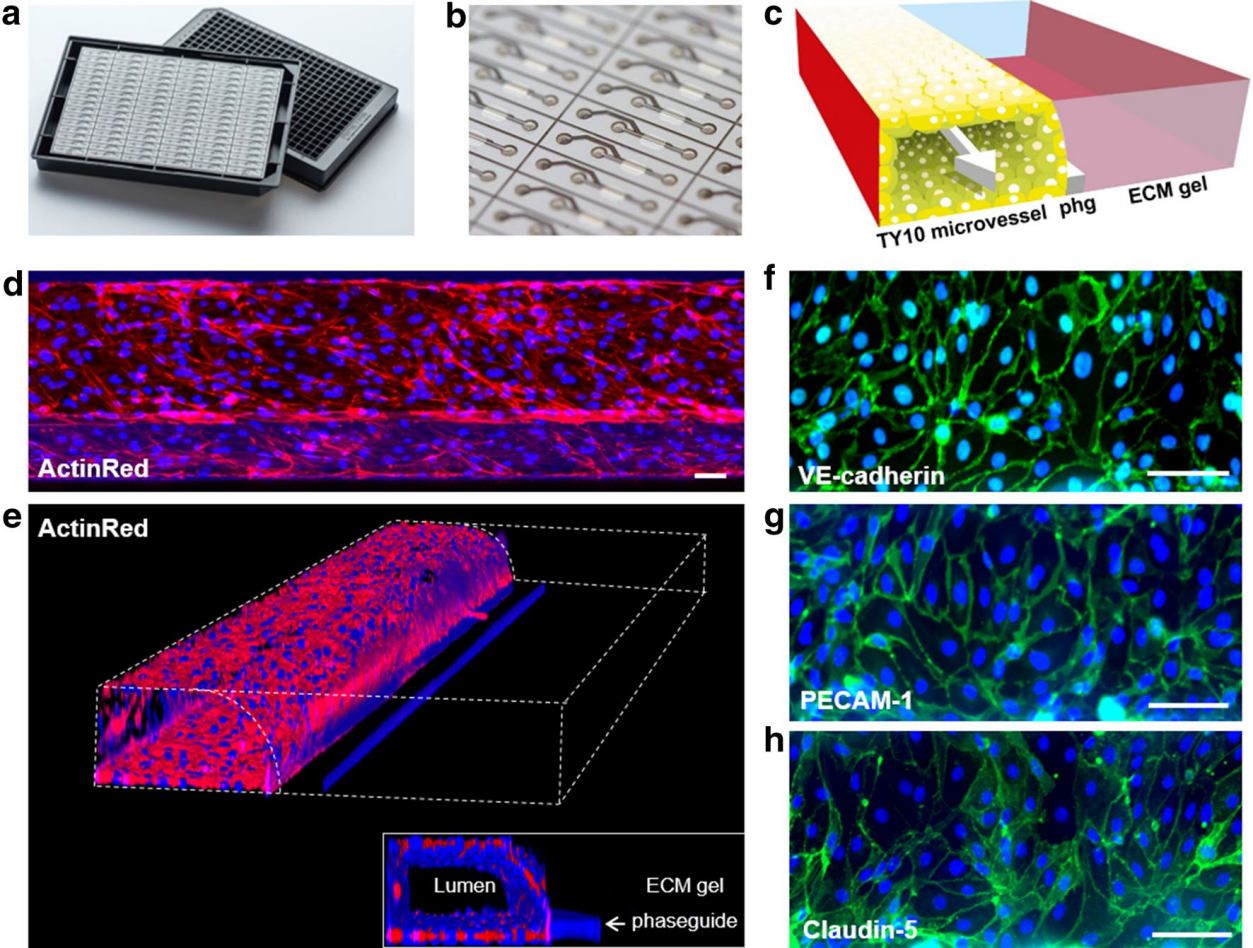
Perfused microvessels of TY10 brain endothelial cells in the two-lane OrganoPlate. **a** Picture shows the two-lane OrganoPlate. The plate combines a 384-well microtiter plate on the top with microfluidic channels on the bottom that make up 96 tissue culture chips. **b** Zoom-in of the bottom of the two-lane OrganoPlate, showing the tissue culture chips that consist of two channels: a gel channel and a medium channel. **c** 3D artist impression of the center of a chip. ECM gel is added to the gel channel and a phaseguide (phg) prevents it from flowing into the adjacent medium channel. After gelation of the ECM gel, TY10 cells are added to the medium channel and a TY10 microvessel forms. The microvessel has a lumen at its apical side that is perfused. **d** Maximum projection image of a TY10 microvessel stained with ActinRed. Scale bar is 50 µm. **e** 3D reconstruction of a confocal z-stack showing a perfused TY10 microvessel. Inlay shows a vertical cross section, depicting the lumen, the phaseguide, and the ECM gel channel. **f**–**h** Immunostaining of a TY10 microvessel for VE-cadherin, PECAM-1, and claudin-5. Scale bar is 50 µm

The two-lane OrganoPlate (see Fig. 1a, b) allows parallel culture of 96 miniaturized tissues on microfluidic tissue chips [40–42]. In each chip, a microvessel of brain endothelial cells was grown under perfusion against an extracellular matrix gel (see Fig. 1c–e and Additional file 1). A 3D reconstruction of a TY10 microvessel stained with ActinRed is shown in Fig. 1e and demonstrates that a vessel-like structure of endothelial cells has formed. Immunostaining was performed to assess the expression of key BBB adherens junction and tight junction proteins (see Fig. 1f–h). TY10 microvessels showed expression of vascular endothelial cadherin (VE-cadherin), platelet endothelial cell adhesion molecule 1 (PECAM-1), and claudin-5 at the cell–cell contacts as expected. The expression and interendothelial localization of these markers are indicative of barrier formation [2, 43].

### Assessment of barrier function in TY10 brain endothelial microvessels

**Fig. 2:**
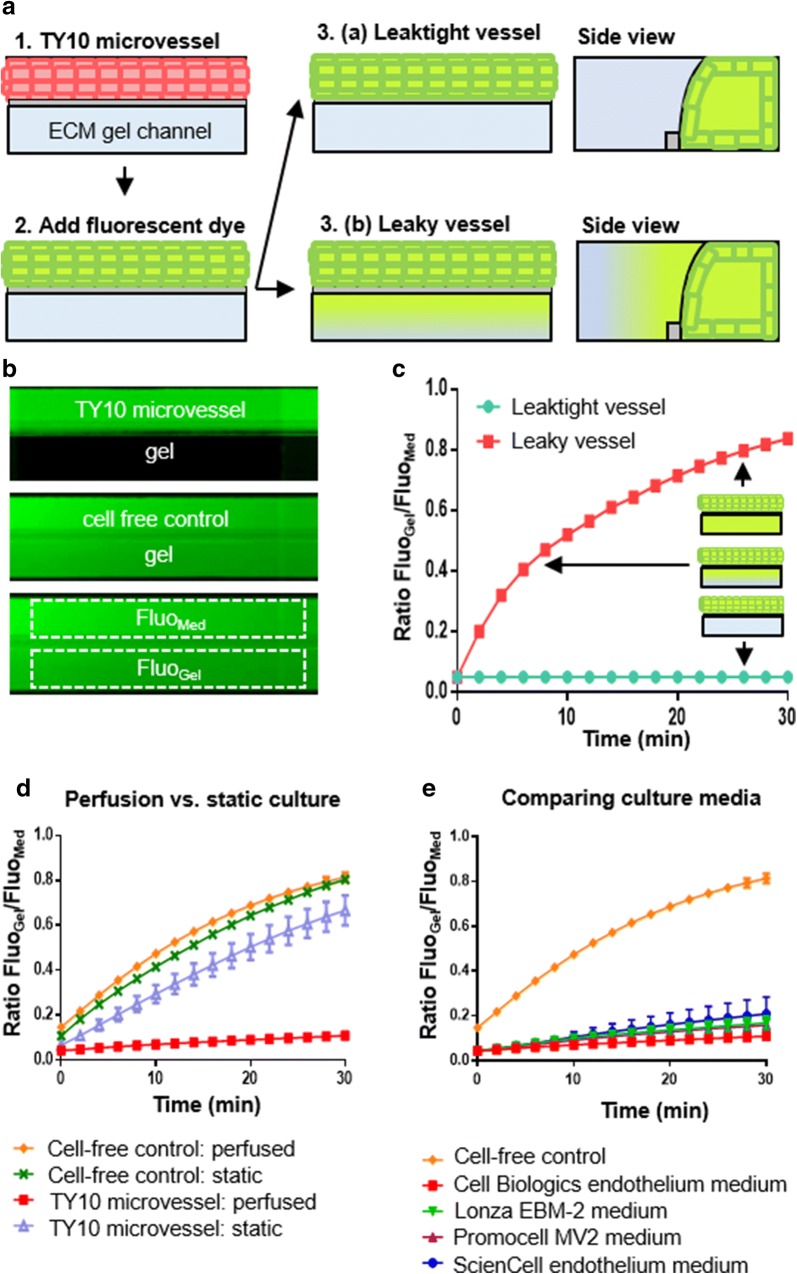
Assessment of barrier function in TY10 brain endothelial microvessels. **a** Schematic representation of the barrier integrity assay. A perfused TY10 microvessel is grown against an ECM gel. A fluorescent dye is added to the medium inlets and outlets and perfused through the lumen of the microvessel. In case of a leaktight vessel, all dye is retained in the vessel. In case of a leaky vessel, the dye leaks into the adjacent gel channel. **b** Fluorescent dye distribution for a TY10 microvessel (top image) and a cell-free control (middle image) during a barrier integrity assay (FITC-dextran, 20 kDa, t = 30 min). To quantify leakage of the fluorescent dye in a chip, the fluorescence intensity measured in the medium channel and the gel channel (Fluo_Med_ and Fluo_Gel_, respectively) are measured over time (bottom image). **c** For each condition the ratio between the fluorescence signals measured in the two channels is plotted over time. In case of a leaktight TY10 microvessel, the ratio Fluo_Gel_/Fluo_Med_ is constant, resulting in a flat horizontal line. In case of a leaky microvessel or a cell-free control, the fluorescence signal measured in the gel channel increases over time, resulting in an increase in the ratio Fluo_Gel_/Fluo_Med_. **d** TY10 microvessels were grown under perfusion or static conditions for 7 days, after which barrier integrity assays (for FITC-dextran, 20 kDa) were performed. n = 6 for TY10 microvessels, n = 2 for cell-free controls. Error bars show standard deviation of the mean. **e** Assessment of barrier function (for FITC-dextran, 20 kDa) of TY10 microvessels cultured under perfusion for 7 days in various commercially available cell culture media. n = 7 for all conditions. Error bars show standard deviation of the mean

The barrier function of TY10 microvessels grown in the OrganoPlate was assessed using a fluorescent barrier integrity assay. TY10 microvessels were perfused with a 20 kDa FITC-dextran dye, which has a hydrodynamic radius slightly smaller than a folded IgG antibody (3 nm [44] versus 5–6 nm [45], respectively). Leakage of the dye from the microvessel into the adjacent gel channel was assessed by acquisition of fluorescent images over time (see Fig. 2a). In a chip containing a leaktight TY10 microvessel, all fluorescent dye is retained within the vessel (see Fig. 2b, top image). In a cell-free control chip, the fluorescent dye can freely diffuse into the adjacent gel channel (see Fig. 2b, middle image). To quantify leakage of the fluorescent dye, areas were selected automatically for each chip to compare over time (see Fig. 2b, bottom image). The ratio of the fluorescence signal measured in the gel channel and the medium channel is plotted over time as an average of all chips within a condition (see Fig. 2c). In chips that contain a tight cellular barrier, this ratio remains constant, as all dye is retained in the microvessel that is grown in the medium channel. In leaky vessels and cell-free controls, the ratio increases over time, as dye leaks into the gel channel. We compared barrier integrity of TY10 microvessels cultured under static conditions to microvessels cultured under perfusion (by passive leveling using an interval rocker). TY10 cells cultured under perfusion show much tighter barrier formation (Fig. 2d) resulting from increased proliferation and elevated expression and improved localization of junctional proteins (see Additional file 3). In addition, we assessed four different commercially available endothelial cell culture media and found that for this specific type of brain microvascular endothelial cell, the endothelial cell medium from Cell Biologics was optimal for barrier formation (see Fig. 2e).

### BBB co-cultures of brain endothelium, astrocytes, and pericytes in the three-lane OrganoPlate

**Fig. 3:**
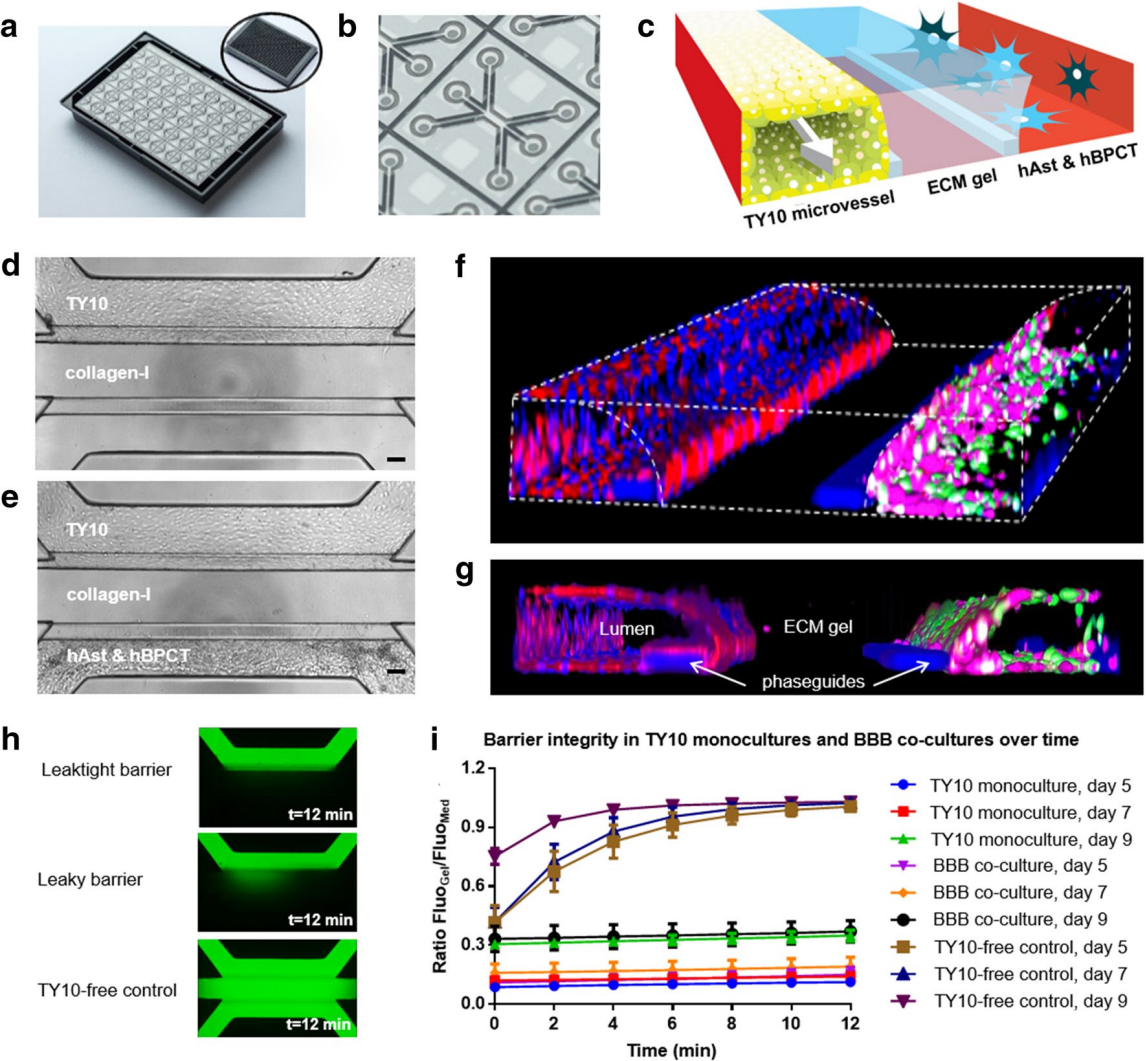
BBB co-cultures of brain endothelium, astrocytes, and pericytes in the three-lane OrganoPlate. **a** Picture shows the three-lane OrganoPlate. The plate combines a 384-well microtiter plate on the top with microfluidic channels on the bottom that make up 40 tissue culture chips. **b** Zoom-in of the bottom of the three-lane OrganoPlate, showing one tissue culture chip that consists of three channels. **c** 3D artist impression of the center of a chip. ECM gel is added to the gel channel and two phaseguides (white rims) prevent it from flowing into the adjacent medium channels. After gelation of the ECM gel, TY10 cells are added to one of the medium channels and allowed to form a vessel structure. The microvessel has a lumen at its apical side that is perfused. Next, astrocytes (hAst cells) and pericytes (hBPCT cells) are added to the other medium channel to establish a BBB co-culture. **d**, **e** Phase contrast images of a TY10 monoculture (top image) and a BBB co-culture (bottom image) in the three-lane OrganoPlate, day 7. Scale bar is 100 µm. **f**, **g** 3D reconstruction of a confocal z-stack showing the organization of the three cell types in a BBB co-culture. The hAst and hBPCT cells were labeled with green-fluorescent calcein-AM and calcein red™ AM, respectively, here depicted in the colors green and magenta. Tight junctions of the TY10 microvessel are shown by a PECAM-1 staining (red). **h** Images acquired of chips with a leaktight barrier (top), a leaky barrier (middle), or TY10-free control (gel with hAst and hBPCT, bottom) during a barrier integrity assay (for FITC-dextran, 20 kDa, t = 12 min). **i** Assessment of barrier function (for FITC-dextran, 20 kDa) at different time points for TY10 monocultures and BBB co-cultures cultured under perfusion in the three-lane OrganoPlate. n = 3 for TY10-free controls and TY10 monocultures and n = 6 for BBB co-cultures. Error bars show standard deviation of the mean

While endothelial cells make up the brain’s vasculature, other cell types, such as astrocytes and pericytes, are also part of the BBB and help maintain barrier function. A three-lane OrganoPlate [46] (see Fig. 3a, b) was employed to establish a BBB co-culture of TY10 brain endothelial cells with hAst and hBPCT cells (see Fig. 3c, f, g and Additional file 2). A barrier integrity assay (see Fig. 3h) was performed to determine barrier function in TY10 monocultures (Fig. 3d) and BBB co-cultures (Fig. 3e) at different time points during culture. Both TY10 monocultures and BBB co-cultures successfully retained, to a great extent, a 20 kDa FITC-dextran dye in the endothelial microvessel at days 5, 7, and 9 as is apparent by the marginal increase in the ratio of fluorescence signal measured in the gel channel and the medium channel over time (see Fig. 3i). This barrier integrity assay is useful in identifying optimal culture conditions and timing for which to perform antibody transcytosis assays and showed minimal passive permeability of molecules that are similar in size to antibodies.

### Assessment of antibody transcytosis across the BBB

**Fig. 4:**
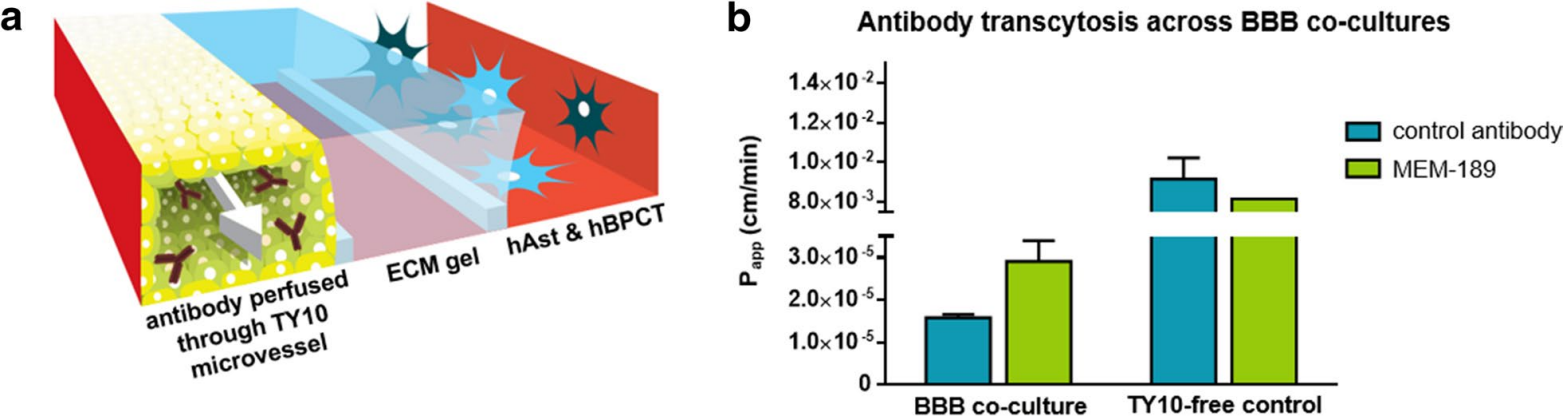
Receptor-mediated transcytosis of antibodies across the BBB on-a-chip model. **a** 3D artist impression of the assessment of antibody transcytosis across BBB co-cultures in the three-lane OrganoPlate. Antibody is perfused through the lumen of the TY10 microvessel. Samples are taken from the apical compartment (ECM gel channel and channel in which hAst and hBPCT cells are grown). **b** Apical samples taken from BBB co-cultures and TY10-free controls were analyzed and apparent permeability (P_app_) of control antibody anti-HEL and target antibody MEM-189 are plotted. n = 2–6 for BBB co-cultures and n = 1–2 for TY10-free controls. Error bars show the standard deviation of the mean

Two different antibodies were used to assess their passage across our human in vitro BBB-on-a-chip. The first was MEM-189, an antibody that binds the human transferrin receptor (hTfR), which is expressed by TY10 endothelial cells (see Additional file 4a) and has been reported to undergo RMT [47]. Flow cytometry analysis showed that anti-hTfR MEM-189 bound to TY10 cells and that binding was not blocked by 25 µg/mL transferrin [EC_50_ = 0.44 ± 0.09 nM (−Tf); 0.5 ± 0.1 nM (+Tf)], see Additional file 4b). The second antibody, anti-hen egg lysozyme (anti-HEL), does not bind a target on human cells and served as a negative control [48]. Both antibodies showed the expected molecular weight, heavy-light chain composition, and size in solution for properly folded antibodies. The antibodies were perfused through the lumen of BBB co-cultures in the three-lane OrganoPlate for 1 h, after which samples were collected from the basal side of the chips (see Fig. 4a). Antibody concentrations in basal samples were determined using MesoScale Discovery^®^ MULTI-ARRAY^®^ technology and the apparent permeability (P_app_) was calculated. Similar basal concentrations of both antibodies were measured in samples taken from chips without a TY10 microvessel, indicating that when no barrier is present, both antibodies diffuse through the gel to an equal extent (see Fig. 4b). However, in BBB co-cultures, passage of antibody MEM-189 was markedly higher than passage of the control antibody (2.9 ×   10^−5^ versus 1.6 × 10^−5^ cm/min, respectively) (see Fig. 4b).

## Discussion

This study describes a significant advancement in the development of a novel BBB model that incorporates human brain endothelial cells, astrocytes, and pericytes in a high-throughput microfluidic platform that can be used for screening purposes. The cells used in this model are immortalized and thus their phenotype may differ from cells of the BBB in living organisms. Therefore, results obtained in this model may not directly apply to patients. However, the TY10 brain endothelial cell line used in this study has been shown to express relevant junctional markers and transporters, independent of the passage number [31]. Although primary brain endothelial cells offer an interesting alternative, they are difficult to obtain reliably from human donors and have been described to rapidly dedifferentiate and lose their BBB characteristics after removal from the in vivo environment, resulting in decreased expression of essential BBB modulators and impaired barrier function [49, 50]. In recent years, tremendous progress has been made in the generation of induced pluripotent stem cell (iPSC)-derived brain endothelial cells. These cells have been shown to express relevant junctional proteins and transporters, respond well to cues from supporting cell types such as astrocytes and pericytes, and display in vivo-like barrier properties [51–55]. In addition, a recent report showed that iPSC-derived brain endothelial cells could be used to study antibody transcytosis across the BBB [55]. The combination of our high-throughput, membrane-free, perfused platform with the ongoing advancements in iPSC-derived cell types may potentially bring about highly relevant BBB models in the future.

In contrast to the standard 2D Transwell approach [19, 56–58], the OrganoPlate supports perfused culture of brain endothelial cells, which was shown to be essential for proper lumen formation, proper expression and localization of junctional proteins, and improved barrier function (see Fig. 2d and Additional file 3). Perfusion is generated by placing the entire plate on a rocker platform, allowing medium to flow from medium inlet to outlet and back, creating a bidirectional flow. Fluid flow is controlled by regulating the inclination angle and the interval with which the rocker platform switches sides. Although direct in vivo measurements of shear stress in BBB vessels and venules of different diameters are lacking, it is likely that the shear stress established in our model (~ 1.2 dyne/cm^2^) is low compared to the shear stress experienced by vessels of similar diameter and curvature in vivo [59, 60]. Several reports have described improved barrier function of brain endothelial cells as a result of increased shear stress due to an upregulation in junctional proteins [61–63]. Interestingly, other studies did not find increased expression of junctional proteins, but report that brain endothelial cells exhibit a unique response to shear stress compared to endothelium in different organs. Unlike other endothelial cell types, brain endothelial cells did not elongate or align in response to shear stress, a phenotype that may be associated with the BBB’s unique properties [64, 65]. The setup used in this study bypasses the need for pumps and intricate tubing systems, which are often associated with microfluidic culture systems, and improves the user-friendliness and throughput of the method.

Comparison of barrier integrity in different culture platforms is challenging, as the properties of the platform itself often influence the measured outcome and are difficult to properly correct for. Among these properties are the presence or lack of a membrane, the pore size of the membrane, the volumes used for the assay, the presence of flow, and the nature of the read-out. Standard measures such as a barrier’s transelectrical endothelial resistance (TEER) or a compound’s P_app_ can be used to compare results obtained within the same platform, i.e. a Transwell, but cannot directly be compared to results obtained in other culture platforms. The observation that the BBB on-a-chip successfully limits the passage of molecules of similar size as antibodies shows that sufficient barrier function is established to investigate antibody passage. In addition, Trietsch et al. [46] have reported higher sensitivity in detection of compound-induced CaCo-2 barrier disruption in the OrganoPlate compared to Transwell. The increased sensitivity was expected to result from improved maturity of the culture as well as a decreased dead volume and higher surface-to-volume ratio in the OrganoPlate.

Many microfluidic systems employ polydimethylsiloxane (PDMS) because of its optical properties and ease of use in the fabrication process. However, PDMS has several limitations in organ-on-a-chip applications. The intrinsic hydrophobicity of the material impedes cell adhesion and can cause non-specific absorption of proteins and hydrophobic analytes. Although several methods are available to reduce PDMS hydrophobicity and fouling problems, the most successful method requires a complex process that is difficult to incorporate for large scale production [66, 67]. The OrganoPlate employs optical quality (170 µm) glass and polymers that are biocompatible and low compound-absorbing and does not include an artificial membrane. Furthermore, the collagen gel does not significantly restrict passage of antibody into the basal compartment, which was demonstrated by the observation that antibody P_app_ increased > 500-fold when the endothelial cells were omitted from the model (Fig. 4b). Together, the low level passive permeability of the brain endothelial cell layer, minimal absorption, and the ease of antibody sampling from the basal compartment could support sensitive and high-throughput screening of antibody transcytosis in this BBB on-a-chip.

The human transferrin receptor is of special interest for drug targeting to the brain due to its expression on brain endothelial cells and potential to support receptor mediated transcytosis [9, 12, 68, 69]. In this study, we observed an approximately two-fold higher passage of MEM-189, an antibody targeting the hTfR receptor, across our BBB model compared to a control antibody. The P_app_ value we measured for the non-binding control antibody agrees well with those reported recently in other in vitro human BBB models [55, 70]. The passage of this murine IgG control antibody could in theory result from Fc receptor mediated transport through the neonatal Fc receptor (FcRn), which is expressed at the BBB. However, since murine IgG1 antibodies show very little binding to the human FcRn [71] and FcRn likely does not result in BBB transcytosis [72], the passage of this antibody is most likely the result of paracellular flux or non-receptor mediated endocytic flux (i.e. micropinocytosis). The enhanced permeability of the MEM-189 antibody is consistent with previous reports [47] and could result from active transport mediated by the transferrin receptor. This bivalent antibody has high affinity for hTfR and thus is not optimized for high transport, as has been shown for other anti-TfR BBB shuttles [9, 12, 15]. As a large portion of endocytosed TfR has been shown to remain in the endothelial cells instead of undergoing transcytosis, it is likely that a relatively large quantity of endocytosed MEM-189 remains within the endothelial cells [73]. However, Sade et al. [47] have shown that binding of MEM-189 to the TfR is pH-dependent, which may support transcytosis via release from the TfR in the endosomal environment and could contribute to its observed transcytosis. The assay developed in this study is sensitive to differences in antibody passage across the BBB model and holds potential to be applied in larger screens for discovery of CNS-penetrant antibodies. The addition of neurons to the model may add an extra layer of complexity that brings about possibilities for modelling the full neurovascular unit and studying the passage of molecules across the BBB as well as the effects of these molecules on neuronal function.

## Conclusion

In summary, the model described here is the first perfused BBB-on-a-chip culture system that is compatible with standard laboratory equipment and allows throughput to an extent that is necessary for drug screening. Moreover, co-culture complexity is easily expanded as shown by the addition of pericytes and astrocytes. This model could support further discovery and engineering of antibody BBB-shuttle technologies.

## Additional files

**Additional file 1.** Endothelial microvessel seeding in the two-lane OrganoPlate. (**a**) Schematic representation of one chip of a two-lane OrganoPlate. (**b**) An ECM gel is seeded in the gel channel, after which endothelial cells are seeded in the medium channel. (**c**) Endothelial cells attach to the ECM gel and perfusion is started by placing the OrganoPlate on a rocker platform. (**d**) A microvessel of endothelial cells is formed. (**e**–**g**) Cross sectional view of steps described in **b**–**d**.

**Additional file 2.** BBB co-culture seeding in the three-lane OrganoPlate^®^. (**a**) Schematic representation of one chip of a three-lane OrganoPlate. (**b**) ECM gel is seeded in the middle gel of the chip, after which endothelial cells (TY10) are seeded in the top channel. (**c**) Endothelial cells attach to the ECM and perfusion is started by placing the plate on a rocking platform. (**d**) A microvessel of endothelial cells forms in the top channel, against the ECM gel. (**e**) Astrocytes (hAst) and pericytes (hBPCTs) are seeded in the bottom channel. (**f**) hAst and hBPCT cells attach and a BBB co-culture is established. (**g**–**k**) Cross sectional view of steps described in **b**–**f**.

**Additional file 3.** Comparing perfused and static culture of TY10 microvessels. (**a**, **b**) Phase contrast images of TY10 microvessels grown in the two-lane OrganoPlate under perfused or static conditions (day 7). Scale bar is 100 µm. (**c**) Microvessels grown under perfused or static conditions were fixed and nuclei were stained with Hoechst. The average number of nuclei was counted in both conditions and normalized to the perfused condition. n = 6, Student’s t-test p < 0.05. (**d**–**f**) Immunofluorescent staining of TY10 microvessels grown under perfusion for adherens and tight junction markers VE-cadherin, claudin-5, and PECAM-1. (**g**–**i**) Immunofluorescent staining of TY10 microvessels grown static for adherens and tight junction markers VE-cadherin, claudin-5, and PECAM-1. Scale bar is 100 µm.

**Additional file 4.** Characterization of the human transferrin receptor in TY10 endothelial cells. (**a**) Immunofluorescent staining of the hTfR in TY10 endothelial cells. Scale bar is 50 µm. (**b**) Flow cytometry analysis of cell surface binding of anti-TfR MEM-189 to TY10 endothelial cells in the presence and absence of transferrin (25 µg/mL), EC_50_ = 0.44 ± 0.09 nM (−Tf); 0.5 ± 0.1 nM (+Tf).

## Abbreviations

BBB: blood–brain barrier
CNS: central nervous system
FcRn: neonatal Fc receptor
FCS: fetal calf serum
hAst: human astrocyte cell line
hBPCT: human brain pericyte cell line
HEL: hen egg lysozyme
HEPES: 4-(2-hydroxyethyl)-1-piperazineethanesulfonic acid
hTfR: human transferrin receptor
iPSC: induced pluripotent stem cell
MEM-189: anti-transferrin receptor antibody
PBS: phosphate buffered saline
PDMS: polydimethylsiloxane
PECAM-1: platelet endothelial cell adhesion molecule 1
RMT: receptor-mediated transcytosis
TEER: transendothelial electrical resistance
TY10 cell line: human brain endothelial cell line
VE-cadherin: vascular endothelial cadherin
