# Supplementary figures and images for: A perfused human blood–brain barrier on-a-chip for high-throughput assessment of barrier function and antibody transport
Source: Fluids Barriers CNS. 2018 Aug 31;15:23. doi: 10.1186/s12987-018-0108-3 (PMC6117964; doi:10.1186/s12987-018-0108-3)

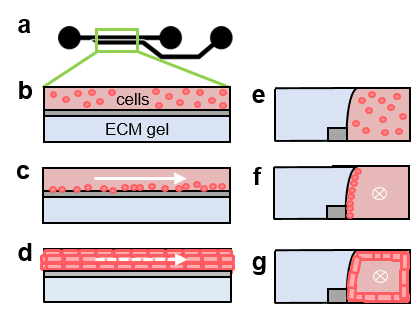

Supplement: Supplementary file 1 — Additional file 1. Endothelial microvessel seeding in the two-lane OrganoPlate. (a) Schematic representation of one chip of a two-lane OrganoPlate. (b) An ECM gel is seeded in the gel channel, after which endothelial cells are seeded in the medium channel. (c) Endothelial cells attach to the ECM gel and perfusion is started by placing the OrganoPlate on a rocker platform. (d) A microvessel of endothelial cells is formed. (e–g) Cross sectional view of steps described in b–d. [file 12987_2018_108_MOESM1_ESM.png]

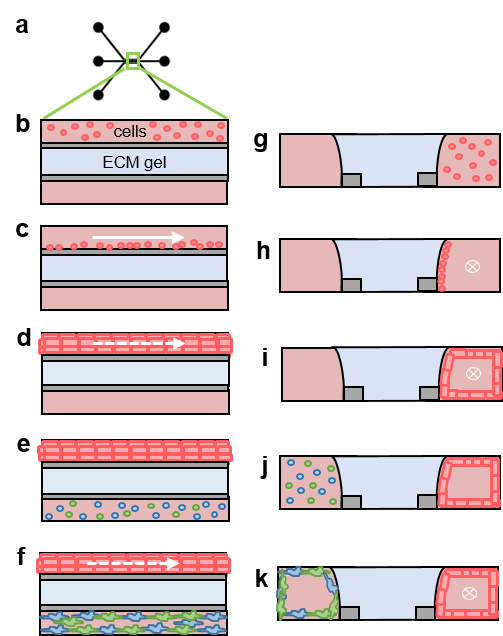

Supplement: Supplementary file 2 — Additional file 2. BBB co-culture seeding in the three-lane OrganoPlate®. (a) Schematic representation of one chip of a three-lane OrganoPlate. (b) ECM gel is seeded in the middle gel of the chip, after which endothelial cells (TY10) are seeded in the top channel. (c) Endothelial cells attach to the ECM and perfusion is started by placing the plate on a rocking platform. (d) A microvessel of endothelial cells forms in the top channel, against the ECM gel. (e) Astrocytes (hAst) and pericytes (hBPCTs) are seeded in the bottom channel. (f) hAst and hBPCT cells attach and a BBB co-culture is established. (g–k) Cross sectional view of steps described in b–f. [file 12987_2018_108_MOESM2_ESM.png]

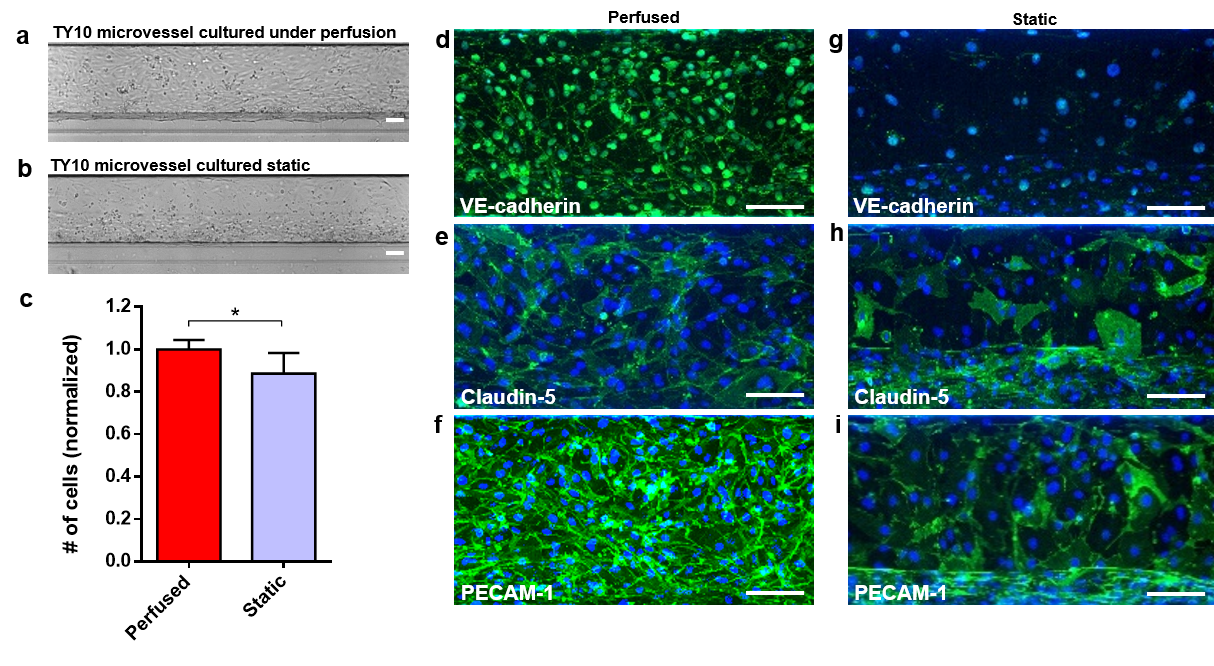

Supplement: Supplementary file 3 — Additional file 3. Comparing perfused and static culture of TY10 microvessels. (a, b) Phase contrast images of TY10 microvessels grown in the two-lane OrganoPlate under perfused or static conditions (day 7). Scale bar is 100 µm. (c) Microvessels grown under perfused or static conditions were fixed and nuclei were stained with Hoechst. The average number of nuclei was counted in both conditions and normalized to the perfused condition. n = 6, Student’s t-test p < 0.05. (d–f) Immunofluorescent staining of TY10 microvessels grown under perfusion for adherens and tight junction markers VE-cadherin, claudin-5, and PECAM-1. (g–i) Immunofluorescent staining of TY10 microvessels grown static for adherens and tight junction markers VE-cadherin, claudin-5, and PECAM-1. Scale bar is 100 µm. [file 12987_2018_108_MOESM3_ESM.png]

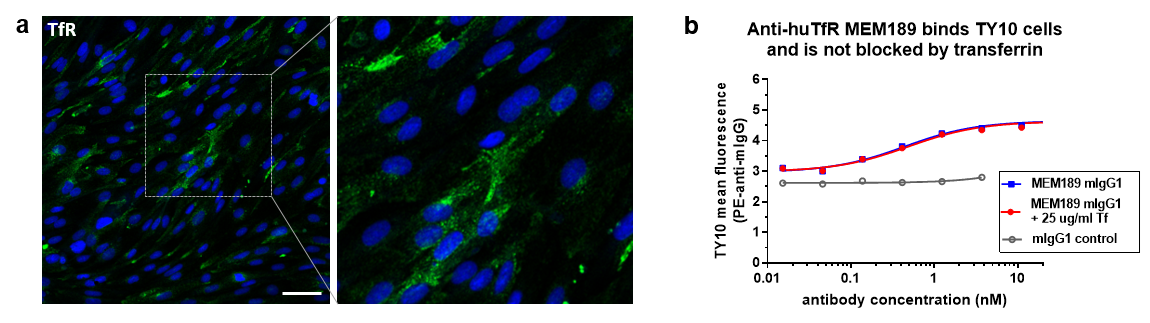

Supplement: Supplementary file 4 — Additional file 4. Characterization of the human transferrin receptor in TY10 endothelial cells. (a) Immunofluorescent staining of the hTfR in TY10 endothelial cells. Scale bar is 50 µm. (b) Flow cytometry analysis of cell surface binding of anti-TfR MEM-189 to TY10 endothelial cells in the presence and absence of transferrin (25 µg/mL), EC50 = 0.44 ± 0.09 nM (−Tf); 0.5 ± 0.1 nM (+Tf). [file 12987_2018_108_MOESM4_ESM.png]
